# Comprehensive Characterization of a Novel Broad-Host-Range Lytic *Salmonella* Phage WP110 and Its Biocontrol Potential Across the Broiler Value Chain

**DOI:** 10.3390/antibiotics15080747

**Published:** 2026-07-31

**Authors:** Wattana Pelyuntha, Wichanan Wannasrichan, Haemarat Khongkhai, David Yembilla Yamik, Mingkwan Yingkajorn, Vincent Guyonnet, Kitiya Vongkamjan

**Affiliations:** 1 Futuristic Science Research Center, School of Science, Walailak University, Thasala, Nakhon Si Thammarat 80160, Thailand; wattana.pe@wu.ac.th; 2 Research Center for Theoretical Simulation and Applied Research in Bioscience and Sensing, Walailak University, Thasala, Nakhon Si Thammarat 80160, Thailand; 3 Department of Biotechnology, Faculty of Agro-Industry, Kasetsart University, Chatuchak, Bangkok 10900, Thailand; wichanan.wan1991@gmail.com (W.W.); haemarat.k@gmail.com (H.K.); davidyembilla.y@ku.th (D.Y.Y.); 4 Department of Pathology, Faculty of Medicine, Prince of Songkla University, Hat Yai, Songkla 90110, Thailand; mingkwan.y@psu.ac.th; 5 Département de Sciences Cliniques, Faculté de Médecine Vétérinaire, Université de Montréal, Saint-Hyacinthe, QC J2S 2MC, Canada; vincent.guyonnet18@gmail.com

**Keywords:** bacteriophage, biocontrol, broiler, endolysin, genome characterization, peptidoglycan, *Salmonella*

## Abstract

Background/Objectives: *Salmonella enterica* (*S. enterica*) is a major poultry-associated foodborne pathogen and a persistent public health concern. The global rise in antimicrobial resistance has accelerated the search for alternative control strategies, including the use of bacteriophages. However, their successful application requires a comprehensive evaluation of their biological performance, genomic safety, and functional proteins. This study aimed to characterize *Salmonella* phage WP110 and assess its potential as a biocontrol agent in broiler-associated production systems. Methods: Phage WP110 was evaluated against 251 *S. enterica* isolates from broiler-related sources. Adsorption kinetics, one-step growth, environmental stability (temperature and pH), and effective multiplicity of infection (MOI) were determined using *Salmonella* Kentucky S1H28. Whole-genome sequencing (WGS) and bioinformatic analyses were performed for genome annotation, taxonomic classification, and safety evaluation. In addition, protein structural prediction of a putative endolysin (WP110-gp057) was conducted using AlphaFold2, followed by structural comparison and molecular docking with peptidoglycan. Biocontrol efficacy was evaluated in contaminated rice husk, chicken meat, and on non-food materials. Results: Phage WP110 demonstrated a broad lytic spectrum, lysing 248/251 *S. enterica* isolates (98.8%). It adsorbed rapidly (within 3–15 min) to host cells and exhibited a latent period of ~20 min with a burst size of 134 particles per infected cell. Phage WP110 remained stable at 4–45 °C and pH 5–11 but was inactivated at ≥75 °C and pH 2. Complete bacterial inactivation in broth assay was achieved at an MOI of 10^4^. Genomic analysis revealed a 110,216 bp linear dsDNA genome (39.74% GC) comprising 204 ORFs, 25 tRNAs, and long direct terminal repeats, with no detectable antibiotic resistance genes. Phylogenetic and intergenomic analyses classified phage WP110 as a novel species within the genus *Epseptimavirus*. Structural modeling of WP110-gp057 revealed conserved catalytic residues and high structural similarity to T5 endolysin, while docking analysis supported a structurally plausible interaction with peptidoglycan at the predicted active-site groove, consistent with its proposed role in host cell wall degradation. In application models, phage WP110 significantly reduced *Salmonella* contamination in rice husk (up to 4.3 log CFU/g), chicken meat (up to 1.7 log CFU/g), and on non-food material surfaces (0.7–1.5 log CFU reduction). Conclusions: Phage WP110 is a broad-host-range lytic phage with favorable infection kinetics, environmental robustness, and genomic safety. Its functionally supported endolysin and strong antibacterial efficacy across broiler-associated matrices highlight its potential as a biocontrol agent for *Salmonella* mitigation in poultry value chain.

## 1. Introduction

*Salmonella enterica* (*S. enterica*) is one of the most important foodborne bacterial pathogens worldwide and is responsible for a wide range of clinical manifestations collectively referred to as salmonellosis. This disease creates a substantial public health and economic burden in both developed and developing countries. According to the World Health Organization [1], non-typhoidal *Salmonella* causes millions of infections annually, resulting in significant morbidity and mortality globally. In 2019, more than half a million cases of invasive non-typhoidal *Salmonella* disease (iNTS) illnesses were diagnosed. Of these, more than 79,000 were estimated to have led to death, with the majority occurring in countries in sub-Saharan Africa [2]. In the US, the Centers for Disease Control and Prevention (CDC) estimates approximately 1.35 million illnesses, 26,500 hospitalizations, and 420 deaths each year, with direct medical costs exceeding USD 400 million [3].

Food-producing animals, particularly poultry, pigs, and cattle, serve as major reservoirs and transmission vehicles of *Salmonella* along the farm-to-fork continuum, facilitating the persistence and dissemination of this pathogen within food production value chains [4,5,6,7]. Among these, broiler chickens are recognized as one of the most critical sources of contamination due to the intensive nature of poultry production and the increased risk of bacterial spread during rearing, slaughtering, and processing. Consequently, contaminated poultry meat and poultry-derived products represent a major route of human *Salmonella* infection worldwide [8]. Furthermore, inadequate hygiene practices during food handling, processing, and preparation can exacerbate cross-contamination events and significantly increase the risk of foodborne outbreaks [9].

The prevalence of *Salmonella* in poultry farms varies considerably across geographical regions, reflecting differences in farm management, biosecurity practices, and surveillance systems. Epidemiological studies have demonstrated a wide range of contamination levels, from less than 2% for some broiler flocks to more than 30% in others. Various surveys conducted in African poultry production systems have demonstrated a variable but persistent detection of *Salmonella* across farms, indicating that this pathogen remains endemic in primary production environments [10,11,12,13]. The continued incidence of *Salmonella* at both on-farm and processing stages highlights the limitations of existing control measures. Traditionally, antibiotics have been extensively used in animal production for growth promotion, disease prevention and treatment. However, the widespread and often indiscriminate use of antimicrobials has accelerated the emergence and dissemination of multidrug-resistant *Salmonella* strains, posing a serious threat to both animal and public health [14]. Growing concerns regarding antimicrobial resistance have consequently prompted regulatory agencies to restrict or prohibit the use of antibiotics as growth promoters in food animals, underscoring the urgent need for effective alternative strategies to control *Salmonella* in poultry production systems [15,16].

The broiler value chain encompasses a series of interconnected production stages, including grandparent and parent stock farms, hatcheries, broiler grow-out farms, transportation, slaughterhouses, processing plants, distribution, and retail. Throughout this continuum, *Salmonella* may be introduced, persist, or spread via contaminated feed, litter, equipment, processing environments, or cross-contamination during slaughter and meat handling. Consequently, effective control strategies should ideally be applicable across multiple stages of the broiler value chain to reduce bacterial dissemination and improve food safety.

Bacteriophages (phages) are bacterial viruses specifically infecting and lysing bacteria that have re-emerged as promising biocontrol agents for combating foodborne pathogens in food production systems [17,18]. Phages exhibit high host specificity, self-replication at the site of infection, and minimal impact on beneficial microbiota, enabling a targeted reduction in pathogens without disrupting commensal microbes [19]. Several recent studies have demonstrated the efficacy of phage-based interventions in reducing *Salmonella* contamination in poultry and food products, both in model systems and in commercial settings [20]. For example, novel *Salmonella* phages with potent lytic activity have been characterized for use in various poultry environments, underscoring their potential role in enhancing food safety protocols [21,22].

Despite their promising potential as biocontrol agents, the successful application of phages requires the comprehensive characterization of their biological properties, host range, genomic features, and safety profile to ensure the absence of undesirable genes, including virulence or antimicrobial resistance determinants. The WGS has therefore become an essential step for assessing the safety and practical suitability of candidate phages for food and agricultural applications.

Several studies have reported the isolation and application of *Salmonella* phages in broiler value chain, including the use of phage cocktails to reduce *Salmonella* colonization and bacterial loads in broilers [23,24,25]. In particular, phage WP110 was previously identified as a highly lytic candidate and was incorporated into phage cocktail formulations that demonstrated reductions in *Salmonella* in both in vitro and in vivo tests [26,27]. However, the detailed genomic characterization of this phage has not yet been performed, and its stability under individual environmental stresses, such as varying temperature and pH conditions, has not been systematically evaluated. Moreover, the efficacy of phage WP110 as a single-phage application for potential commercial use in broiler production systems remains unexplored. These knowledge gaps hinder the comprehensive evaluation of its safety, stability, and practical application, particularly for its use as a standalone intervention in poultry value chain.

In the present study, phage WP110 was comprehensively characterized to evaluate its potential as a biocontrol agent against *Salmonella* in the broiler value chain. The study combined biological characterization, including host range determination, efficiency of plating (EOP), adsorption kinetics, one-step growth analysis, thermal and pH stability, and MOI assays, with WGS and bioinformatic analyses to assess its genomic features, taxonomy, and safety. In addition, the structure of a putative endolysin was predicted using AlphaFold2 and analyzed by molecular docking to investigate its potential function. Finally, the biocontrol efficacy of phage WP110 was evaluated in representative broiler-associated matrices, including rice husks, chicken meat, and non-food contact surfaces. Together, these approaches provide a comprehensive assessment of the biological properties, genomic safety, and practical applicability of phage WP110 for controlling Salmonella throughout the broiler value chain.

## 2. Results

### 2.1. Lytic Activity of Salmonella Phage WP110

In a previous study, phage WP110 was isolated from a wastewater sample obtained from an aerated pond at the wastewater treatment facility of Prince of Songkla University Hospital. The phage was reported to produce small, clear plaques approximately 0.1 mm in diameter on a lawn of *S.* Kentucky S1H28. Transmission electron microscopy analysis revealed an icosahedral head and a long tail. Based on these morphological characteristics, phage WP110 was classified as a member of the *Siphoviridae* family within the order *Caudovirales* [23]. To evaluate the host specificity of phage WP110, its lytic activity was examined against a collection of 251 *S. enterica* isolates representing different sources throughout the broiler value chain using the spot assay.

The lytic spectrum analysis showed that phage WP110 exhibited 100% lytic activity against *Salmonella* isolates obtained from grandparent stock farm (G; *n* = 1), feed mills (F; *n* = 4), parent stock farms (P; *n* = 61), hatcheries (H; *n* = 41), and SL broiler processing plant (*n* = 38). Additionally, the phage lysed 96.6% of isolates from broiler farms (B; 56/58 isolates) and 97.9% of isolates from the SP broiler processing plant (47/48 isolates). Overall, phage WP110 lysed 248 out of 251 *Salmonella* isolates, corresponding to a lytic efficiency of 98.8% (Table 1).

### 2.2. Efficiency of Plating of Salmonella Phage WP110 on Representative Salmonella Isolates

The EOP was subsequently determined for representative isolates to confirm productive infection and differences in phage propagation efficiency. The EOP of phage WP110 against *Salmonella* isolates from various sources (Table 2). Most isolates exhibited high susceptibility, with EOP values > 0.5. The original isolation and propagation host, *S.* Kentucky S1H28, was used as the reference host and assigned a normalized EOP of 1.00. Several isolates exhibited EOP values > 1, indicating higher relative plating efficiency than that observed on *S.* Kentucky S1H28. High EOP values were observed in isolates from grandparent stock farms (G1; 2.03 ± 0.06), feed mills (F4; 1.37 ± 0.12), parent stock farms (0.77 ± 0.17 to 2.71 ± 0.23), hatcheries (0.63 ± 0.04 to 1.21 ± 0.17), and processing plants (SL: 1.46 ± 0.22 to 3.11 ± 0.31; SP17 and SP26: 0.69 ± 0.06 to 1.81 ± 0.14). In contrast, variability was noted in broiler farm isolates, where B8 was inefficient (<0.001) and B38 showed low EOP (0.03 ± 0.007), while B53 remained high (0.88 ± 0.04). Similarly, SP3 showed inefficient infection (<0.001). Overall, phage WP110 demonstrated broad infectivity against most *Salmonella* isolates, with only a few showing low or inefficient susceptibility.

### 2.3. Phage Adsorption Assay and One-Step Growth Curve of Salmonella Phage WP110

**Figure 1:**
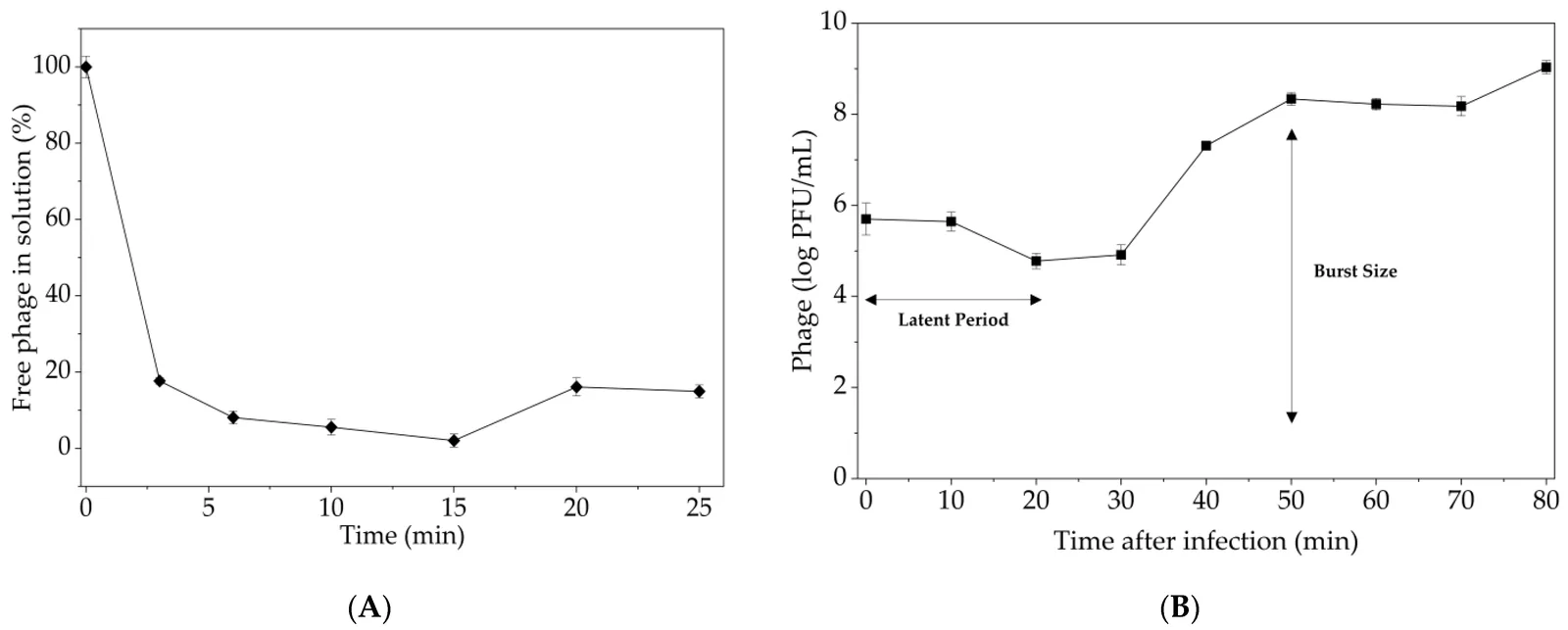
Adsorption assay of *Salmonella* phage WP110 from infection with natural host *Salmonella* Kentucky S1H28. The percentage of free phages was calculated as the ratio of PFU in the supernatant to the initial PFU, using equivalent dilutions of the phages without host cells as references (**A**). One-step growth of *Salmonella* phage WP110 following infection with natural host *Salmonella* Kentucky S1H28 at MOI of 0.01 in TSB at 37 °C (**B**). Values represent the means of triplicate, with error bars indicating the standard deviations.

Phage adsorption kinetics were assessed by quantifying free and adsorbed phages over 25 min. No adsorption was detected at 0 min (100% free). Rapid adsorption occurred within 3 min (82.35%), increasing to 91.96% at 6 min and peaked at 94.51–97.98% between 10 and 15 min, with free phages reaching a minimum of 2.02%. After 20 min, adsorption declined to 83.92%, likely reflecting the release of phages due to host lysis or detachment (Figure 1A). Overall, the results indicated rapid initial adsorption, maximal binding at 3–15 min, and subsequent progression of the infection cycle.

One-step growth analysis of phage WP110 revealed a latent period of approximately 20 min, during which phage titers remained relatively constant at 4 log PFU/mL. Following this phase, a sharp increase in phage concentration was observed between 20 and 40 min, indicating the onset of the rise period and active release of progeny phages. The phage titer subsequently reached a plateau at approximately 8 log PFU/mL after 50 min. Based on the increase in phage count, the average burst size was estimated at 134 particles per infected cell (Figure 1B).

### 2.4. Thermal and pH Stability of Salmonella Phage WP110

**Figure 2:**
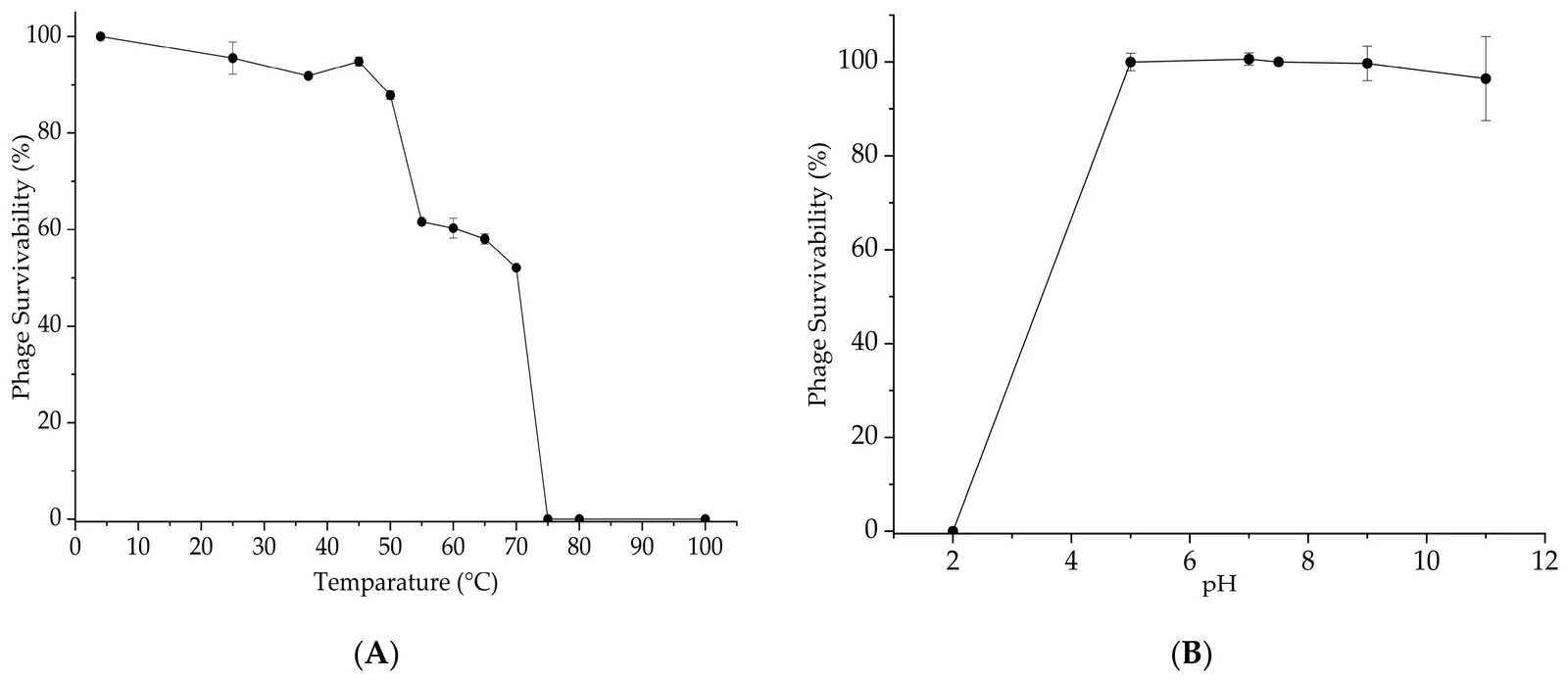
Thermal (**A**) and pH (**B**) stability of *Salmonella* phage WP110.

Phage stability was assessed by plaque assay after exposure to different temperatures and pH conditions. The phage remained highly stable between 4 and 45 °C, with infectivity gradually decreasing above 50 °C and completely inactivated at ≥75 °C (Figure 2A). The phage also exhibited broad pH stability (pH 5–11) but was completely inactivated at pH 2 (Figure 2B).

### 2.5. Effective Multiplicity of Infections (MOIs) of Salmonella Phage WP110

To determine the effective MOI of phage WP110, *Salmonella* counts were enumerated after 6 h of treatment at different MOIs (Table 2). A significant reduction of 0.5 log units (from 7.0 ± 0.3 to 6.5 ± 0.8 log CFU/mL) was observed at MOI 10^1^ compared with the control (*p* < 0.05), whereas MOI 10^0^ resulted only in a 0.2 log reduction, without statistical significance (*p* > 0.05). Complete inactivation was achieved at MOIs 10^4^ and 10^5^, identifying MOI 10^4^ as the minimum effective MOI. Phage titers varied during the assay, as shown in Table 3.

### 2.6. Genome Characteristic of Salmonella Phage WP110 and Its Classification

**Figure 3:**
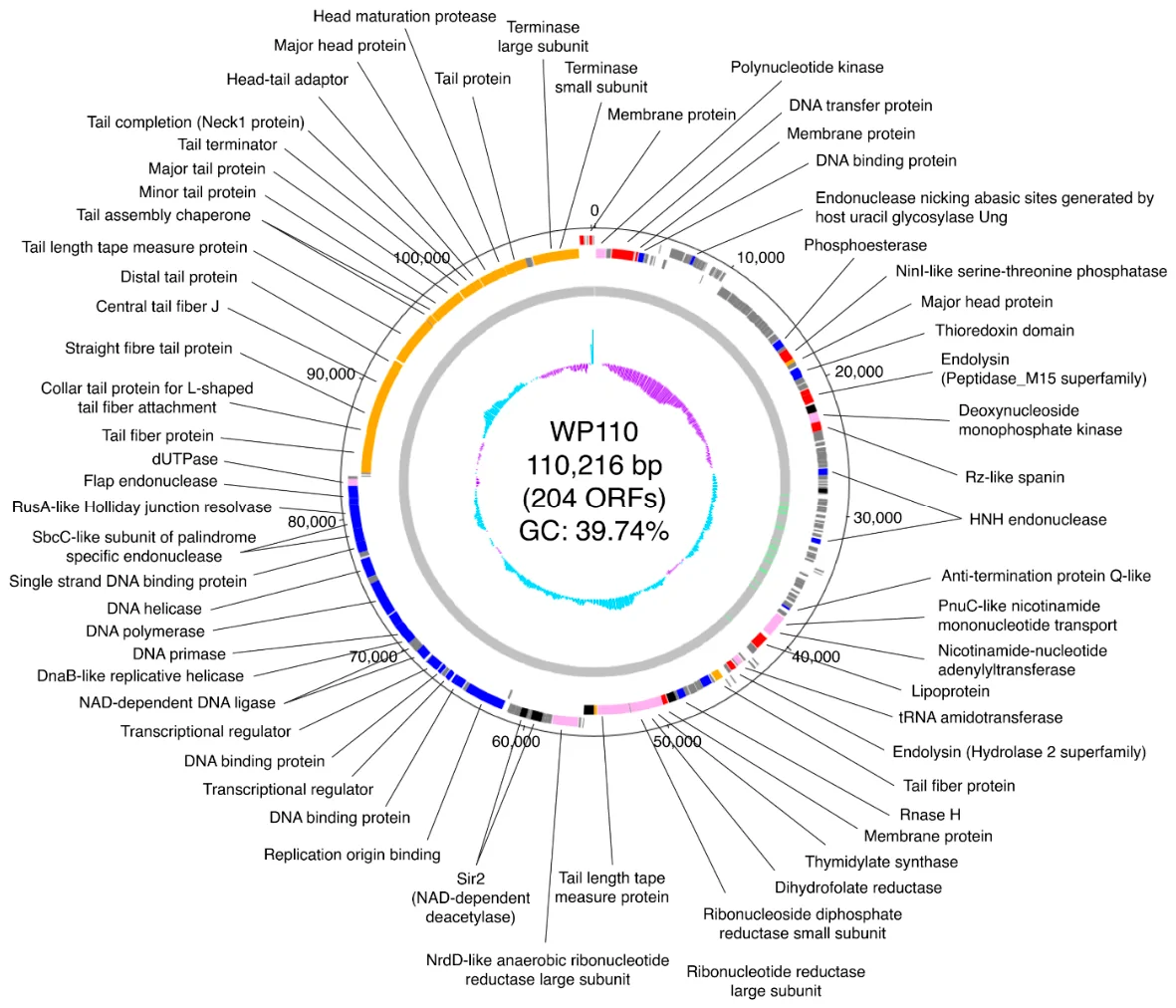
Circular genomic map of phage WP110. ORFs indicated by colors as follows: blue, DNA replication, transcription and translation and DNA repair; pink, nucleotide metabolism and modification; orange, virion structural and assembly; red, host-phage interaction and host lysis protein; black, others; and grey, hypothetical proteins. Green stripes on the middle-grey circle represent the loci of tRNA. An inner plot presents the GC content across the genome (light blue above average and magenta below average). The numbers around the map represent nucleotides. The map was created using Artemis: DNAPlotter version 18.1.0.

The complete genome of phage WP110 was obtained through WGS. The genome topology of phage WP110 was determined as a linear double-stranded DNA molecule, which was not permuted but terminally redundant with long direct terminal repeats (DTRs) (Figure S1). The genome size was estimated at 110,216 bp with a GC content of 39.74%, and 204 open reading frames (ORFs) predicted (Figure 3). Among all predicted ORFs, 126 were annotated as hypothetical proteins, whereas functional predictions were assigned to 78 ORFs involved in following biological processes such as: DNA replication, transcription and translation, and DNA repair (25 ORFs); nucleotide metabolism and modification (14 ORFs); virion structural components and assembly (23 ORFs); host–phage interaction and host lysis (12 ORFs); and other functions (4 ORFs). Interestingly, 25 tRNA loci were identified in the phage WP110 genome, encoding tRNAs for isoleucine (GAT), threonine (TGT), glycine (TCC), glutamine (TTG), glutamine (CTG), arginine (ACG), histidine (GTG), leucine (TAG), alanine (TGC), alanine (TGC), valine (TAC), lysine (TTT), methionine (CAT), proline (TGG), glycine (GCC), lysine (CTT), aspartic acid (GTC), asparagine (GTT), cysteine (GCA), phenylalanine (GAA), tryptophan (CCA), glutamic acid (TTC), leucine (TAA), methionine (CAT), and arginine (TCT). Moreover, an antibiotic resistance gene was not found in the WP110 genome (Table S1).

**Figure 4:**
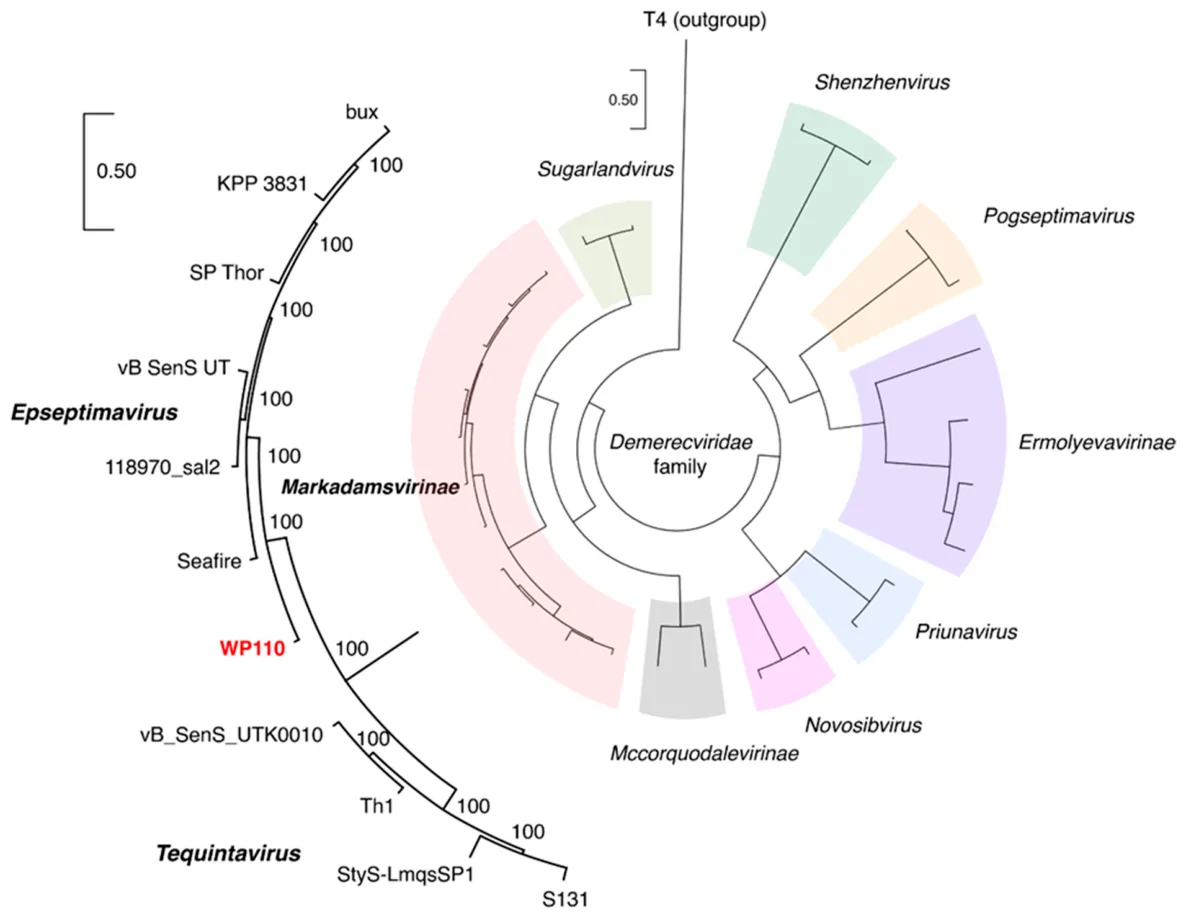
Phylogenetic analysis revealed that phage WP110 is a member of *Epseptimavirus* genus in the *Markadamsvirinae* subfamily, *Demerecviridae* family. A circular tree was constructed by comparing WP110 with 26 genomes (RefSeq) of phage members of the *Demerecviridae* family and using phage T4 (NC_000866.4) as an outgroup. The alignment was performed with MAFFT version 7, and the tree was constructed with Maximum Likelihood (ML) method with 100x bootstrap (http://atgc.lirmm.fr/phyml/ accessed on 1 April 2026). The numbers on the branches represent bootstrap values (%), and the scale bar indicates the branch distance between taxa. The accession numbers of phages used in the phylogenetic tree are as follows: *Markadamsvirinae* (NC_104259.1, NC_048009.1, NC_134262.1, NC_048795.1, NC_103484.1, NC_111488.1, and NC_133303.1, NC_048110.1, NC_092684.1, and NC_031933.1); *Sugarlandvirus* (NC_041899.1, and NC_042093.1) *Mccorquodalevirinae* (NC_047887.1, and NC_018837.1); *Ermolyevavirinae* (NC_055851.1, NC_042094.1, NC_047882.1, and NC_019529.1); *Pogseptimavirus* (NC_048041.1, and NC_048151.1); *Shenzhenvirus* (NC_047949.1, and NC_047950.1); *Novosibvirus* (NC_042090.1, and NC_048086.1); and *Priunavirus* (NC_055910.1, and NC_041913.1).

To genetically classify phage WP110, a BLASTn (version 2.17.0) analysis was first performed using its genome sequence against genome sequences available in the NCBI database. The results indicated that the genome sequence of phage WP110 was similar to those of phages belonging to the *Demerecviridae* family. Therefore, a phylogenetic tree was constructed using the genome sequence of phage WP110 and representative phages from each genus within this family. The phylogenetic analysis revealed that phage WP110 was clustered with a group of *Salmonella* phages, namely bux (NC_092684.1), KKP_3831 (NC_133303.1), SP_Thor (NC_103484.1), vB_SenS_UTK0010 (NC_111488.1), 118970_sal2 (NC_031933.1), and Seafire (NC_048110.1). These phages are all members of the genus *Epseptimavirus* within the *Markadamsvirinae* subfamily, indicating that phage WP110 should be classified as a member of the genus *Epseptimavirus* (Figure 4).

**Figure 5:**
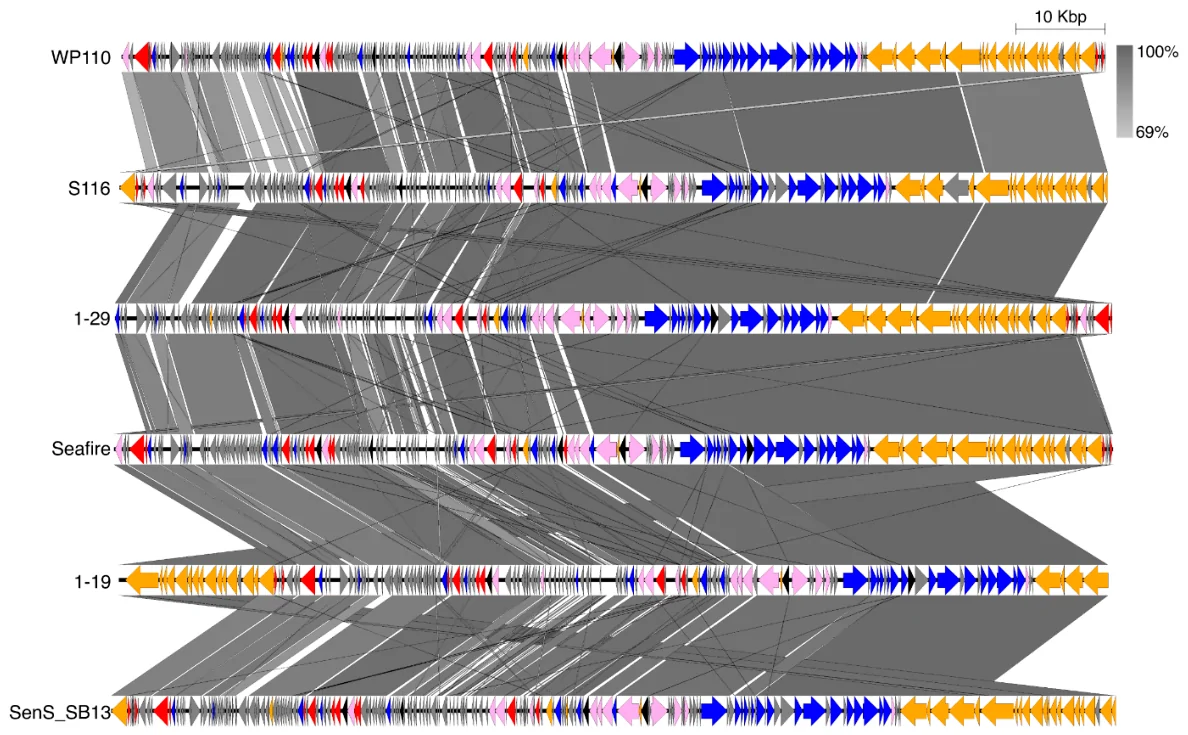
Comparative genomic analysis revealed that phage WP110 exhibits similar genome organization to its closest relatives in *Epseptimavirus* genus. The ORF patterns of phage WP110 was compared to those of S116 (NC_048007.1), 1–29 (NC_048811.1), Seafire (NC_048110.1), 1–19 (NC_048819.1), and SenS_SB13 (NC_048781.1). Scale bar represents 10 kilobase pairs and similarity between ORFs varies from 65% (light grey) to 100% (dark grey). Colored arrows represent ORFs: DNA replication, transcription and translation and DNA repair (blue); nucleotide metabolism and modification (pink); virion structural and assembly (orange); host-phage interaction and host lysis protein (red); others (black); and hypothetical proteins (gray).

Nucleotide similarities between phage WP110 and all 56 *Salmonella* phage members (RefSeq) of the genus *Epseptimavirus* were subsequently analyzed using VIRIDIC. The VIRIDIC analysis revealed that the genome sequence of phage WP110 was most closely related to S116 (NC_048007.1) with 88.27% identity, followed by 1–29 (NC_048811.1) with 88.12%, Seafire (NC_048110.1) with 87.71%, 1–19 (NC_048819.1) with 87.46%, and SenS_SB13 (NC_048781.1) with 87.49% (Table S2). These results indicated that phage WP110 represents a novel species within the genus *Epseptimavirus*, according to the demarcation criteria established by the ICTV specifying that members of the same phage species must share more than 95% genome sequence identity [28,29]., The genome of phage WP110 was compared with those of S116, 1–29, Seafire, 1–19, and SenS_SB13 to further investigate its relationship with closest relatives. The comparison revealed that although the overall genome organization of phage WP110 was similar to that of these phages in terms of ORF arrangement, certain genes with the same predicted functions differed in size, particularly those within the cluster associated with DNA replication, transcription and translation, and DNA repair (blue) (Figure 5).

### 2.7. Protein Structural Prediction and Docking Analysis of WP110-gp057 Endolysin

**Figure 6:**
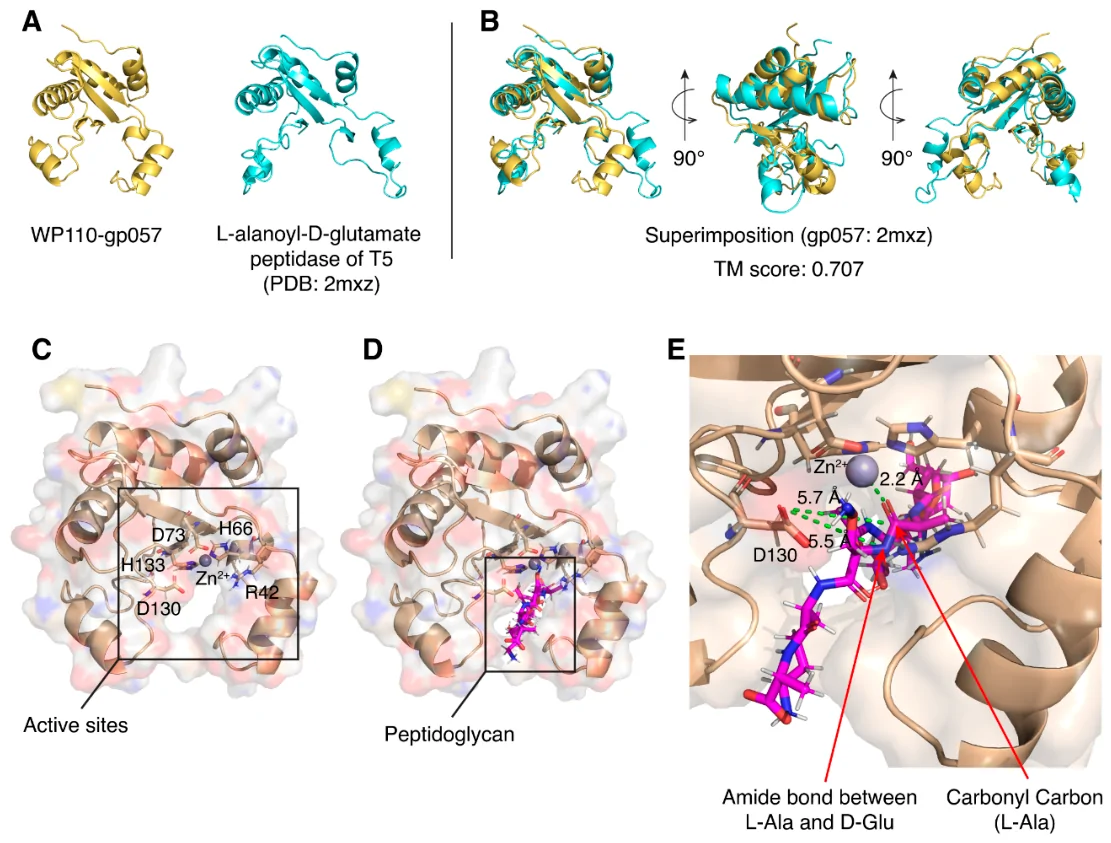
3D structure of WP110-gp057 contained similar conserved domains to L-alanoyl-D-glutamate peptidase (PDB: 2mxz) as T5 phage with the same set of active site residues. (**A**) 3D structure of WP110-gp057 simulated by Alphafold2 compared to L-alanoyl-D-glutamate peptidase (PDB: 2mxz) of T5 phage endolysin. (**B**) Superimposition between the structure of WP110-gp057 and the T5 endolysin run by TM align server. (**C**) A black frame shows potential active sites (R42, H66, D73, D130, and H133) and Zn+2 in the WP110-gp057 structure surrounding the pocket cavity. (**D**) A black frame shows a molecule of peptidoglycan (PubChem SID: 223440619) docked at the pocket cavity surrounded by the potential active sites. (**E**) Close-up view of the docking between WP110-gp057 and the peptidoglycan by SWISSDOCK shows the proximity between active residues of WP110-gp057 and the peptidoglycan. A green dash represents the distance (Å) of possible interactions between Zn^2+^ and a carbonyl oxygen of L-Alanine; between a carbonyl oxygen of D130 and an amide bond (between L-Alanine and D-Glutamate), and the carbonyl carbon of L-Alanine.

Beyond the genome analysis, the potential functions of proteins involved in key roles during phage infection were investigated. In addition to WP110-gp057, ORF84 and ORF142 were also annotated as putative endolysins; however, WP110-gp057 was selected for further structural analysis because of its homology to the well-characterized T5 endolysin. NCBI conserved domain analysis indicated that WP110-gp057, predicted as an endolysin, contained L-ala-D-glu peptidase domains (L-alanyl-D-glutamate endopeptidase). Five putative active residues (R42, H66, D73, D130, and H133) were identified. Consistently, Phyre2 revealed structural similarity to the T5 phage endolysin (endopeptidase class), which hydrolyzes the L-alanine–D-glutamic acid bond in *E. coli* peptidoglycan [30,31]. A 3D model was generated using AlphaFold2 (ColabFold), and the top-ranked structure was selected for analysis (Figure S2). Predicted template modelling score (pTM) of the WP110-gp057 structure is equal to 0.678 as a pTM above 0.5 means the overall predicted fold for the complex might be similar to the true structure. WP110-gp057 exhibited conserved domains highly similar to those of T5 endolysin (PDB: 2mxz), with a TM-score of 0.707 (Figure 6A,B), suggesting functional similarity. The active residues were localized around a groove, proposed as the substrate-binding site (Figure 6C). Given the Zn^2+^ dependence of T5 L-alanyl-D-glutamate endopeptidase [30,31], Zn^2+^ was incorporated to model the holoenzyme [32]. The ion was coordinated by H66, D73, and H133, consistent with T5 endolysin [32]. The Zn^2+^-bound structure was docked with peptidoglycan using SwissDock. Among the generated models, model 52 was selected as the most plausible (Table S3), indicating substrate binding within the active-site groove (Figure 6D). Structural analysis showed that Zn^2+^ is positioned ~2.2 Å from the carbonyl oxygen of the L-ala–D-glu amide bond, while D130 is located ~5.7 Å from the carbonyl carbon (Figure 6E).

### 2.8. Effect of Salmonella Phage WP110 on Salmonella-Contaminated Rice Husks

The effective MOI concentration of the phage was applied on rice husks to monitor the reduction in *Salmonella* contamination. As shown in Table 4, there was no significant difference in the *Salmonella* population between the control and phage treatment at the initial time (t = 0). Starting from day 3 of incubation, significant differences (*p* < 0.05) were observed: a 2.6 log unit reduction at day 3, peaking at 4.3 log units on day 5 and slightly decreasing thereafter (days 7 and 14). Phage titer in contaminated rice husks was also monitored via a double-overlay method. It was found that the phage number slightly increased from 6.3 log PFU/g to 6.9 log PFU/g between day 0 and day 3 but dramatically decreased to approximately 4.6 to 5.9 log PFU/g from day 5 to day 14 of the study.

### 2.9. Effect of Salmonella Phage WP110 on Salmonella-Contaminated Chicken Meat

In chicken meat samples, *Salmonella* counts in the phage treatment were significantly reduced by 0.8 log units (*p* < 0.05) at day 1 of storage at 4 °C when compared to the control. On day 3 of storage, phage-treatment significantly reduced *Salmonella* count by 0.7 log units, whereas the maximum reduction (1.7 log units) was observed on day 5. In addition, the phage concentration on chicken meat was also monitored during this 5-day storage period, oscillating between 6.4 and 7.4 log PFU/g (Table 5).

### 2.10. Effect of Salmonella Phage WP110 on Salmonella on Non-Food Material Surfaces

The counts of *Salmonella* were significantly reduced by 1.5, 0.7, and 1.2 log units (*p* < 0.05) after 24 h of phage treatment on ceramic, polyvinyl chloride (PVC), and stainless-steel surfaces, respectively while the control for each surface material maintained a *Salmonella* count of approximately 3 log units (Table 6). In addition, the phage titer for each treatment was approximately 2 to 5 log PFU/cm^2^ during the assay.

## 3. Discussion

The present study demonstrated that phage WP110 possesses a broad lytic spectrum, producing clear lysis against 248 of 251 tested isolates (98.8%) in the spot assay. Complete lysis was observed with isolates from various sources, including breeding farms, hatcheries, and processing plants, thus indicating consistent activity throughout the different stages of the broiler value chain. This level of coverage is relatively high compared to other reports on *Salmonella* phages, often exhibiting narrow host ranges [32,33,34,35]. Phage WP110 was previously isolated from wastewater and shown to infect multidrug-resistant strains and common circulating serovars [23]. The molecular basis underlying the broad host range of WP110 remains unclear. One possible explanation is the recognition of bacterial surface receptors conserved among the tested poultry-associated *Salmonella* isolates; however, the receptors utilized by WP110 were not identified in the present study. Further investigation of its receptor-binding proteins and corresponding host receptors is therefore required to elucidate the mechanisms underlying its broad host range. From a practical application standpoint, such broad lytic activity is particularly advantageous as it reduces the need for complex phage cocktail mixtures and supports the potential use of phage WP110 as a single-phage intervention within poultry value chain.

Differences in EOP among isolates further indicated host-dependent permissiveness, with some strains supporting higher propagation than the original host. Variability in lytic activity and propagation efficiency among *Salmonella* strains has also been observed in other phages recently characterized. For example, the lytic spectrum of phage ISTP3 varied across tested *Salmonella* isolates, indicating strain-dependent susceptibility rather than uniform infectivity across all hosts [36]. A similar host-dependent replication pattern was observed in our previously characterized phage collection, where EOP values differed markedly among isolates and certain strains acted as highly permissive hosts enabling rapid phage amplification [23,37]. From a practical standpoint, the presence of high-EOP hosts is advantageous for large-scale phage production and for achieving efficient bacterial reduction after application.

The adsorption kinetics of phage WP110 showed rapid attachment to the host cells, with more than 80% adsorption occurring within the first 3 min and reaching over 90% within 6–15 min. This rapid binding indicates efficient recognition and interaction with host surface receptors, which is a critical step for successfully invading *Salmonella*. A similar rapid adsorption has been reported for other *Salmonella* phages, with high adsorption efficiency associated with strong lytic performance and effective bacterial control [38,39]. The slight decrease in adsorption observed after 20 min may reflect the onset of the infection cycle, including host cell lysis and the release of progeny phages, rather than its reduced binding ability. In addition, adsorption efficiency can be influenced by the host physiological state and receptor availability, which may affect infection dynamics under different environmental conditions [40]. Overall, the rapid and high adsorption rate of phage WP110 supports its strong infectivity and suggests its suitability for applications requiring quick bacterial targeting.

The one-step growth curve of phage WP110 revealed a short latent period of approximately 20 min, followed by a rapid rise phase and a burst size of 134 particles per infected cell. These parameters indicate the efficient intracellular replication and effective release of progeny phages. Comparable kinetics have been reported for other *Salmonella* phages, such as vB_ntSalS-cftriSP11, exhibiting a similar latent period (~20 min) but slightly higher burst size (~146 PFU per cell) [41], positioning phage WP110 within the expected range of highly lytic phages. A short latent period combined with a relatively high burst size is advantageous for biocontrol applications, as it enables rapid amplification of phage populations and accelerated reduction in bacterial hosts. These characteristics are particularly important in dynamic environments, such as poultry production systems, where fast bacterial growth and contamination occur. Overall, the growth kinetics of phage WP110 supports its strong lytic capacity and further reinforces its potential as an effective *Salmonella* control measure.

Phage WP110 exhibited good stability across a wide range of environmental conditions, remaining active between 4 and 45 °C and within pH 5–11, while complete inactivation was observed at ≥75 °C and pH 2. This stability profile is particularly relevant to the broiler value chain, where environmental conditions vary across stages, from farm environments (ambient temperature and neutral pH) to processing and cold storage conditions. The ability of phage WP110 to remain active under these conditions suggests that it can maintain infectivity throughout the multiple points along the value chain. Temperature and pH are key factors influencing phage performance under practical applications, as they directly affect the phage structure, host interaction, and overall lytic activity. In broiler production systems, phages may be exposed to fluctuating temperatures during rearing, transportation, processing and refrigerated storage, as well as varying pH conditions on various surfaces, feed materials, or meat products. Therefore, the observed tolerance of phage WP110 under moderate temperatures and broad pH ranges supports its potential for applications both in pre-harvest and post-harvest settings. Consistent with previous studies, *Salmonella* phages that maintain stability across diverse physicochemical conditions are more likely to perform effectively within food-related environments [42,43,44]. However, the loss of activity at extreme heat and highly acidic conditions suggests that phage WP110 may be less suitable for processes involving thermal treatments or strong acid exposure. Overall, its stability profile supports its practical use at various stages such as farm-level interventions, processing environments, and refrigerated storage, where conditions are within its tolerance range.

The effectiveness of phage WP110 was strongly influenced by its MOI, with complete bacterial inactivation observed at MOIs of 10^4^ and 10^5^, while lower MOIs resulted in only partial reduction in *Salmonella*. This finding indicates that an adequate phage-to-bacteria ratio is critical to achieve effective bacterial control. MOI is a key parameter in phage applications, as it reflects the likelihood of phage particles successfully encountering and infecting host cells [45]. The improved efficacy observed at higher MOIs in this study is consistent with previous reports showing that higher phage concentrations enhance antibacterial activity by increasing the probability of host infection and rapid bacterial clearance [46,47]. In contrast, low MOIs may result in insufficient phage numbers to effectively control bacterial populations, allowing continued bacterial growth or delaying lysis. In practical terms, this suggests that applying phages at higher concentrations is generally more effective than using low phage doses, particularly in systems with heavy bacterial loads, as supported by the present experimental results. Additionally, high MOI conditions can accelerate the initial bacterial killing by ensuring that a large proportion of cells are infected simultaneously, reducing the time required for phage amplification and population control [48]. However, it should also be noted that the optimal MOI may vary depending on environmental conditions and application systems, and excessively high phage doses may not always be necessary or cost-effective. Overall, the results of this study highlighted that MOI plays a critical role in determining the success of phage WP110 as a biocontrol agent, and that selecting the appropriate phage concentration is essential for achieving efficient *Salmonella* reduction under field conditions.

Among the predicted ORFs in the phage WP110 genome, 25 tRNA genes were identified, collectively corresponding to 18 amino acids. The presence of such diverse set of phage-encoded tRNAs suggests their potential role in supporting efficient protein translation during infection, particularly under conditions where host tRNA availability may be limiting. Notably, tRNAs for serine and tyrosine were not detected, indicating that phage WP110 may rely on host machinery for these amino acids. Phage-encoded tRNAs have been proposed to enhance translation efficiency and help overcome host defense mechanisms, such as anticodon-targeting nucleases, thereby facilitating successful phage replication [49]. In tRNA-rich phages, the selection of specific tRNAs may also reflect the adaptation to host codon usage and resistance to host-induced degradation. Based on its genomic characterization, phage WP110 was classified as a member of the genus *Epseptimavirus*. Comparative genomic analysis showed that phage WP110 shares a conserved genome organization with closely related phages, particularly in functional modules associated with replication, structure, and host lysis, consistent with its placement within T5-like phages. In addition, the linear double-stranded DNA genome with direct terminal repeats further supports its similarity to the T5-like phage architecture. Importantly, no antibiotic resistance genes or virulence-associated determinants were detected in the phage WP110 genome, supporting its genomic safety and suitability for biocontrol applications. The absence of antibiotic resistance genes is a critical prerequisite for the safe application of phages in food production systems because it minimizes the risk of horizontal transfer of antimicrobial resistance determinants to bacterial populations. Current recommendations for phages intended for biocontrol emphasize comprehensive genome screening to ensure that they do not harbor antibiotic resistance or virulence-associated genes. Therefore, the genomic safety profile of phage WP110 further supports its potential for practical application as a biocontrol agent within the broiler value chain.

The AlphaFold-predicted structure of the putative endopeptidase endolysin (WP110-gp057) revealed conserved domains similar to those of the well-characterized T5 endolysin (EndoT5) (Figure 4). WP110-gp057 shares approximately a 97% amino acid sequence identity with EndoT5 and retains all its key catalytic residues (Figure S3), suggesting a conserved enzymatic function. A Zn^2+^ ion is required to interact with the substrate to initiate peptidoglycan hydrolysis. In WP110-gp057, the residues H66, D73, and H133 are predicted to coordinate the Zn^2+^ ion, forming a catalytic center similar to that of EndoT5. The Zn^2+^ ion interacts with the carbonyl oxygen of the amide bond (L-alanine), promoting polarization of the carbonyl group and destabilizing the bond between L-alanine and D-glutamate. Residue D130 is proposed to play a supportive role in stabilizing the transition state of the intermediate molecule [50]. Docking analysis further supported this mechanism, showing that the Zn^2+^ ion was located approximately 2.2 Å from the carbonyl oxygen of the amide bond (L-alanine), which is consistent with the distance reported for EndoT5 (~2.3 Å) (Figure S4) [32,51,52]. Although Asp130 is positioned ~5.7 Å away from the scissile bond and may not directly form a hydrogen bond, it may indirectly contribute via water-mediated interactions under physiological conditions [53,54]. In addition, residue R42 may function as a substrate-binding residue through electrostatic interactions, enhancing the affinity of the enzyme for peptidoglycan [55]. Together, these structural and docking results support the functional role of WP110-gp057 as an active endolysin involved in host cell wall degradation, providing some mechanistic insight into the lytic activity of phage WP110.

The application of phage WP110 across different matrices demonstrated its potential for controlling *Salmonella* along the broiler value chain. On rice husks, often used as bedding in poultry houses, phage WP110 achieved substantial bacterial reduction, particularly during the early incubation period, highlighting its suitability in pre-harvest interventions. Although comparable studies with bedding materials are limited, previous reports have shown that phage applications at the farm level can reduce *Salmonella* colonization and its environmental persistence in poultry systems [24]. This supports the idea that targeting environmental reservoirs, such as litter or bedding, is an effective strategy to interrupt transmission within flocks. It should be noted, however, that the present study evaluated the biocontrol efficacy of phage WP110 in sterilized rice husks rather than its physicochemical stability under actual poultry litter conditions. Commercial poultry litter is considerably more complex, containing ammonia, organic matter, indigenous microorganisms, and other environmental factors that may influence phage persistence and activity. Therefore, further studies under commercial poultry litter conditions are warranted to determine the stability and long-term performance of phage WP110 under practical farm environments. In chicken meat, phage WP110 reduced *Salmonella* counts by up to 1.7 log CFU/g during refrigerated storage. This level of reduction is consistent with previous studies, where phage treatments typically achieved reductions ranging from approximately 0.7 to 1.3 log CFU/g under similar conditions [56,57]. Slightly higher reductions (up to ~2 log units) have been reported when phages were applied as cocktails or combined with other antimicrobial treatments [58]. Thus, the reduction achieved by phage WP110 falls within the range reported for phage-based interventions in poultry meat and demonstrates promising biocontrol activity, particularly considering that phage WP110 was applied as a single phage without additional antimicrobial hurdles. The moderate reduction observed in this study is also consistent with the known limitations of single-phage applications in complex food matrices, where physical barriers and organic matter can restrict phage–host interactions. Nevertheless, direct comparisons among studies should be interpreted cautiously because differences in phage dose, bacterial inoculum, food matrix, treatment duration, and experimental conditions can influence the observed efficacy. Therefore, although the present findings support the potential of phage WP110 for reducing *Salmonella* contamination in chicken meat, further optimization and validation under commercial processing conditions are required to determine whether this level of reduction is sufficient for practical implementation. On non-food material surfaces, phage WP110 achieved reductions of 0.7–1.5 log CFU/cm^2^, demonstrating antibacterial activity against *Salmonella* on ceramic, PVC, and stainless steel. Previous studies have demonstrated that phages can effectively reduce *Salmonella* on food-contact surfaces, although the extent of reduction often depends on surface type, moisture, bacterial attachment, and treatment conditions [21]. The effectiveness of phage WP110 across these different materials suggests its potential as a supplementary sanitation strategy to reduce the risk of cross-contamination in processing facilities. However, the observed reductions were partial, indicating that further optimization may be required for sanitation-related applications. Increasing the phage concentration, optimizing contact time and formulation, or incorporating phage WP110 into a phage cocktail or hurdle-based intervention may further enhance its efficacy. Overall, the performance of phage WP110 across these application models is consistent with the recognition of phages as effective but often partial control agents. While phage cocktails or combined treatments may achieve greater reductions, the ability of phage WP110 to function as a single-phage intervention may offer advantages in terms of simplicity and targeted application. Taken together, these findings support the potential of phage WP110 as a biocontrol agent across multiple stages of the broiler value chain; however, further validation under commercial-scale conditions, including naturally contaminated samples and operational processing environments, is required to determine its efficacy and feasibility for practical implementation.

Although phage WP110 demonstrated promising biocontrol efficacy and favorable genomic safety, several limitations of the present study should be acknowledged. First, the experiments were limited to in vitro characterization and representative broiler-associated matrices, and therefore the efficacy of phage WP110 under commercial poultry production conditions remains to be validated. Second, the present study did not evaluate the potential impact of phage WP110 on the gut microbiota or beneficial commensal bacteria because no in vivo experiments or microbiome analyses were performed. Finally, the stability and persistence of phage WP110 under additional application-relevant conditions, including different salt concentrations, prolonged storage periods, UV exposure, and complex poultry litter conditions involving ammonia, organic matter, and indigenous microbial communities, warrant further investigation. Addressing these limitations in future studies will provide a more comprehensive assessment of the efficacy, ecological safety, and practical applicability of phage WP110 in poultry production systems.

## 4. Materials and Methods

### 4.1. Bacterial Strains Used

The study was conducted following the biosafety regulations for scientific experiments and was approved by the Institutional Biosafety Committee of Walailak University (Ref. No. WU-IBC-67-076). A total of 251 strains of *S. enterica* used in this study were received from the Department of Biotechnology, Faculty of Agro-Industry, Kasetsart University. All strains were previously isolated from broiler farms (B), grandparent (G) and parent (P) stock farms, broiler processing plants (SL and SP), hatcheries (H), and feed mills (F) [59]. All strains were kept in 15% glycerol solution at −20 °C and pre-cultured in Tryptic Soy Broth (TSB) (HiMedia Laboratories Pvt. Ltd., Nagpur, India) at 37 °C for 18 h before studies.

### 4.2. Phage Lysate Preparation

*Salmonella* phage WP110 from our previous studies was selected for evaluation of its lytic activity [23]. *S.* Kentucky S1H28 was used as a natural host for phage WP110 propagation. This bacterium was previously isolated from broiler farm of eastern Thailand [23]. The phage from our stock was used to prepare 10-fold serial dilutions in Salt-Magnesium (SM) buffer. Appropriate dilutions were used to prepare the overlay with the selected host to yield a semi-confluent lysis, then harvested with 5 mL of SM buffer and centrifuged at 6000 rpm for 15 min at 4 °C. The supernatant was filtrated through 0.20 µm syringe filters, and phage lysates were kept at 4 °C. The phage titer was determined by counting plaques on each dilution plate [23,60].

### 4.3. Evaluation of the Lytic Activity of Salmonella Phage WP110 Against Bacterial Isolates

The lytic ability of phage WP110 was determined by a spot test on the bacterial lawn of each *Salmonella* isolate included in this study. Briefly, 20 µL of each phage lysate (8 log PFU/mL) was spotted on the bacterial host lawn. Phage lysis patterns were determined after 18–24 h incubation at 37 °C [58,61]. The host specificity of phage WP110 was defined based on its ability to produce a clear lysis zone on each tested isolate in the spot assay, whereas productive infection was further confirmed by determining the EOP on representative isolates.

### 4.4. Efficiency of Plating of Salmonella Phage WP110 on Representative Salmonella Isolates

The EOP of *Salmonella* phage WP110 was evaluated on representative *Salmonella* isolates following the protocol described by Chanthavong et al. [62]. This study was conducted three times independently using three dilutions (4–6 log PFU/mL) against 18 *Salmonella* isolates. The EOP value was calculated using the given formula:

EOP = average PFU on target bacteria/average PFU on host bacteria

EOP was classified as “high production” when the ratio was 0.5 or more. An EOP of 0.1 or higher, but below 0.5, was considered as “medium production” efficiency, and that between 0.001 and 0.1 was considered as “low production” efficiency. An EOP of 0.001 or below and when any dilutions did not result in any plaque formation were classified as “inefficient”.

### 4.5. Phage Adsorption Assay for Salmonella Phage WP110

To estimate the adsorption time of phage WP110, a bacterial culture (*S.* Kentucky S1H28) at 8 log CFU/mL was infected with phage WP110 at the MOI of 0.01 and incubated at 37 °C. Samples were collected at different incubation time points (0, 3, 5, 10, 15, 20, and 25 min), centrifuged at 15,000 rpm for 30 s, and the supernatant serially diluted in SM buffer. A spot-titer test assay of the dilutions was performed to determine the number of free phage particles in the supernatant. The % free phages were plotted against time (minutes post-infection (mpi)). All experiments were done in triplicate [37,60].

### 4.6. One-Step Growth Curve of Salmonella Phage WP110

To evaluate the one-step growth of the phage, a bacterial culture (*S.* Kentucky S1H28) at OD_600_ ~ 0.4 (8 log CFU/mL) was infected with phage WP110 at an MOI of 0.01 and incubated at 37 °C for 10 min. The cells were centrifuged at 8400 rpm for 2 min, the pellet re-suspended in LB broth and incubated at 37 °C for 2 h. Samples were collected every 10 min, filtered with 0.45 µm syringe filters, and serially diluted in SM buffer, and analyzed using a spot-titer assay to determine the number of free phage particles in the supernatant. The phage concentration (PFU/mL) was plotted against time (minutes post-infection (mpi)). Burst size was calculated from the growth curve as the ratio of average maximum titer after lysis to the average baseline titer during the latent period, and expressed as particles per cell [37,60].

### 4.7. Thermal and pH Stability of Salmonella Phage WP110

Thermal stability of the phage (9 log PFU/mL) was evaluated at different temperatures (4, 25, 37, 45, 50, 55, 60, 65, 70, 75, 80, and 100) after 1 h of incubation. The effect of pH condition on phage (9 log PFU/mL) stability was determined at different pH levels (2, 5, 7, 7.5 (control), 9, and 11) during incubation at 37 °C for 24 h. The phage titer was determined by the double-overlay method. All assays were performed in triplicate [27,63].

### 4.8. Effective Multiplicity of Infections (MOIs)

The effective MOI of phage WP110 was evaluated using *S.* Kentucky S1H28 host according to the study of Sun et al. [64] with minor modifications. A *Salmonella* suspension at the final concentration of 3 log CFU/mL in TSB (20 mL) was mixed with the phage lysate at a final concentration ranging from 3 to 8 log PFU/mL (20 mL) to obtain the serial MOI ranging from 10^0^ to 10^5^. The mixture was incubated at 37 °C for 6 h while shaking at 120 rpm. The culture broth (1 mL) was serially diluted and directly spread on xylose lysine decarboxylase (XLD) agar for counting viable *Salmonella*. The mixture was centrifuged at 6500 rpm for 15 min, the supernatant then filtered immediately by 0.22 µM membrane, and the phage titer assayed by the double-layer method.

### 4.9. Genomic DNA Extraction, Whole-Genome Sequencing and Analysis, Phylogenetic Tree, and Genome Comparison of Salmonella Phage WP110

Briefly, the phage lysate (9 log PFU/mL) was precipitated with 10% (*w*/*v*) polyethylene glycol and 1 M NaCl before resuspension in SM buffer. The host DNA and RNA were then digested with 10 U of DNase I and 0.1 mg/mL of RNase A at 37 °C. DNase activity was inhibited by 20 mM EDTA and the phage capsid was then digested by 0.5 mg/mL of proteinase K and 0.5% sodium dodecyl sulphate (SDS) at 55 °C for 1 h. The phage DNA was extracted using phenol-chloroform-isoamyl alcohol (25:24:1) (Thermo Fisher Scientific, Qiryat Shemona, Israel) before treating with 1/10 volume of 3M sodium acetate and 2 volumes of cold absolute ethanol. The genomic DNA was allowed to precipitate at −20 °C for 1 h followed by centrifugation at 15,000 rpm for 20 min. The DNA pellet was washed with 70% ethanol, centrifuged at 12,7000 rpm for 10 min, and dried before dissolved in TE buffer. DNA quality and quantity were analysed using a NanoDrop 2000 spectrophotometer (Thermo Scientific, Waltham, MA, USA) [60,65].

WGS was carried out using the Illumina Novaseq 6000 (Illumina Inc., San Diego, CA, USA). FASTQ files derived from the sequencing were trimmed with fastp version 0.24.0 [66]. The quality of trimmed sequences was checked with FastQC before being assembled with SPAdes version 3.15.5 [67]. The single contig of the phage genome was annotated with Pharokka version 1.3.2 [68,69]. Functional annotation was performed through BLASTP (version 2.17.0) via DNA master version 5.23.6 [70]. NCBI conserved domains (version 3.21) were additionally used for the annotation. Phage genome termini were identified with Phageterm [71]. The phage genome map was constructed and visualized using Artemis: DNAPlotter version 18.1.0 [72].

To construct a phylogenetic tree, the genomic sequences of phage WP110 (accession no. PX354330.1) was aligned with MAFFT version 7 [73] against representative genomic sequences (RefSeq) of phages in all genera and subfamilies of family *Demerecviridae* accounting for 26 genomes. The T4 phage was used as an outgroup. The tree was then constructed using Maximum Likelihood (ML) method [74] with 100 bootstrap replicates and visualized using MEGA-X. Furthermore, genome sequence similarity between phage WP110 and 56 other *Salmonella* phages (RefSeq) of the *Epseptimavirus* genus was analyzed using VIRIDIC [28]. The genome comparison between phage WP110 and its closest *Epseptimavirus* relatives was performed using EasyFig version 2.1 [75] to observe the relationship between their genome organizations.

### 4.10. Protein Structural Prediction and Docking Analysis of the Endolysin of Salmonella Phage WP110

Using its amino acid sequences, the three-dimensional structure of WP110-gp057 was predicted using ColabFold-AlphaFold2 [76]. The top-ranked model generated by the AlphaFold2 was aligned and superimposed with an endolysin (L-alanoyl-D-glutamate peptidase) from *Escherichia coli* T5 phage [77] through Template Modelling (TM) align server [78,79] to assess structural similarity. Peptidoglycan (PubChem SID: 223440619) was selected as the docking target to computationally analyze the possible endolysin function of WP110-gp057. Protein-ligand docking between WP110-gp057 and peptidoglycan was performed using SwissDock with Attracting Cavities model [80,81,82,83]. All predicted and docked structures were visualized and analysed using PyMOL 3.2.0a (Schrödinger Inc., New York, NY, USA).

### 4.11. Effect of Salmonella Phage WP110 on Salmonella-Contaminated Rice Husks

Fresh rice husks obtained from a commercial broiler farm were autoclaved at 121 °C for 20 min to remove any contamination by microorganisms and dried at 70 °C for 3 days. After sterilization, the rice husks were weighed (50 g) and placed on a sterile stainless tray (W 14 cm × L 20 cm × H 3 cm). A dilution of overnight *S.* Kentucky S1H28 (5 mL) culture at a final concentration of 3 log CFU/mL was sprayed onto rice husks, followed by spraying of phage WP110 at effective MOI concentration (~7 log PFU/mL; 5 mL). The tray was kept in a plastic bag and incubated at ambient temperature (25–28 °C) for 14 days. Five-gram samples, taken on days 0, 1, 3, 5, 7, and 14, were mixed with 45 mL of buffered peptone water. The count of *Salmonella* was determined by direct spreading onto XLD agar plates, while the count of phage number presented on rice husks was determined by the double-overlay method as previously described. In addition, rice husks not sprayed with phage were used as control. All tests were run in triplicates.

### 4.12. Effect of Salmonella Phage WP110 on Salmonella-Contaminated Chicken Meat

Chicken meat portions purchased from supermarket were cut into pieces (~5 × 5 cm^3^) of approximately 10 g. All pieces were soaked in 50 ppm free chlorine solution (Sigma-Aldrich, St. Louis, MO, USA) for 5 min to eliminate any interfering microflora, then soaked and washed with sterile distilled water for 5 min three times to remove free chlorine residue. Chicken meat pieces were subsequently soaked in PBS containing the suspension of *S.* Kentucky S1H28 (3 log CFU/mL), left for 15 min to allow bacterial attachment, and then transferred in a sterile plastic zip-lock bag. A 1 mL of phage at effective MOI (~7 log PFU/mL) was added onto each piece of chicken meat. The artificially contaminated chicken meat with added PBS served as a control. All treatments and controls were stored at 4 °C for 5 days. *Salmonella* was enumerated at day 0, 1, 3, and 5 on XLD agar. Typical colonies of *Salmonella* were observed and counted. In addition, the amount of *Salmonella* phages was also quantified using the double-overlay method as previously described [37,57,59].

### 4.13. Effect of Salmonella Phage WP110 on Salmonella on Non-Food Material Surfaces

Three different types of material, ceramic, PVC, and stainless steel, were included in this study to determine the lytic activity of phage WP110 on *Salmonella* present on the surface of non-food materials by a modified method of [84]. For each type of material, small squares (W 4 cm × L 4 cm) were sterilized by autoclave and dried at 70 °C. After placing each square of material onto a Petri-dish, a *Salmonella* suspension at the concentration of 3 log CFU/mL was added and allowed to dry under a biosafety cabinet for 30 min. Then, a phage suspension in SM buffer at the effective MOI was sprayed (0.5 mL; 7 log PFU/mL) on each square and allowed to dry for 2 h. The squares without phage treatment (sterile PBS) were used in controls. A cotton swab was applied to each square to collect the attached bacteria and phages. The counts of *Salmonella* and phages were evaluated as previously described. All treatments were independently performed in triplicate.

### 4.14. Statistical Analysis

Statistical analysis was performed using SPSS (Version 22.0) of Windows statistics software (SPSS Inc., Chicago, IL, USA). Data on *Salmonella* and phage counts for each experiment were subjected to variance analysis followed by Tukey’s range test. Any difference between the control and treatments was evaluated using the independent-sample *t*-test and was statistically significant at *p*-value of less than 0.05.

## 5. Conclusions

This study demonstrated that *Salmonella* phage WP110 is a promising biocontrol agent for controlling *Salmonella*, with broad lytic activity, favorable infection characteristics, and genomic features supporting its suitability for biocontrol applications. Its ability to reduce *Salmonella* in multiple experimental matrices, together with genomic and structural analyses supporting its lytic phenotype, highlights its potential as a biocontrol agent across broiler-associated matrices. Collectively, these findings support the potential of phage WP110 as a biocontrol agent to enhance food safety within the poultry value chain. However, further studies are required to evaluate its long-term efficacy under commercial farm conditions, the potential development of resistance within bacterial populations, and its performance in large-scale field applications. Finally, formulation strategies, including phage stabilization and delivery systems, as well as synergistic use with other interventions such as phage cocktails and biosecurity measures, should be further investigated to facilitate the practical application of phage WP110 in poultry production systems.

## Figures and Tables

**Table 1:** Lytic ability of *Salmonella* phage WP110 on *Salmonella* isolated from various broiler sources.

Sources	No. of Infected Host/No. of Tested Hosts (% Lytic Ability)
Grandparent stock farm (G)	1/1 (100)
Feed mills (F)	4/4 (100)
Parent stock farms (P)	61/61 (100)
Hatcheries (H)	41/41 (100)
Broiler farms (B)	56/58 (96.6)
Broiler processing plant (SL)	38/38 (100)
Broiler processing plant (SP)	47/48 (97.9)
Total	248/251 (98.8)

**Table 2:** The EOP of *Salmonella* phage WP110 against representative *Salmonella.*

Sources	*Salmonella* Isolates	EOP Value ^1^	Category ^2^
LAB collection	S1H28	1.00 *	High
Grandparent stock farm (G)	G1	2.03 ± 0.06	High
Feed mills (F)	F4	1.37 ± 0.12	High
Parent stock farm (P)	P1	0.77 ± 0.17	High
	P8	1.02 ± 0.11	High
	P28	2.71 ± 0.23	High
Hatcheries (H)	H6	0.63 ± 0.04	High
	H36	1.21 ± 0.17	High
	H41	0.67 ± 0.08	High
Broiler farm (B)	B8	<0.001	Inefficient
	B38	0.03 ± 0.007	Low
	B53	0.88 ± 0.04	High
Broiler processing plant (SL)	SL12	1.46 ± 0.22	High
	SL27	3.11 ± 0.31	High
	SL38	2.74 ± 0.18	High
Broiler processing plant (SP)	SP3	<0.001	Inefficient
	SP17	0.69 ± 0.06	High
	SP26	1.81 ± 0.14	High

^1^ All values provided as mean ± standard deviation of triplicate (*n* = 3). ^2^ EOP value: high production (EOP > 0.5), medium production (0.1 > EOP < 0.5), low production (0.001 < EOP < 0.1), and inefficient production (EOP < 0.001). * EOP was calculated as the phage titer obtained on each test isolate divided by the phage titer obtained on the original isolation and propagation host, *S.* Kentucky S1H28, whose EOP was normalized to 1.00. Thus, EOP values > 1 indicate higher relative plating efficiency on the test isolate than on *S.* Kentucky S1H28.

**Table 3:** Effective multiplicity of infection of *Salmonella* phage WP110 upon treatment at 6 h.

Treatment	*Salmonella* Count (log CFU/mL) *	Phage Count (log PFU/mL)
Control	7.0 ± 0.3 ^a^	-
MOI		
10^0^	6.8 ± 0.0 ^a^	2.1 ± 0.7 ^b^
10^1^	6.5 ± 0.3 ^b^	1.7 ± 0.6 ^ab^
10^2^	5.2 ± 0.4 ^c^	1.4 ± 0.1 ^a^
10^3^	3.8 ± 0.2 ^d^	1.3 ± 0.2 ^a^
10^4^	ND	1.2 ± 0.4 ^a^
10^5^	ND	1.2 ± 0.3 ^a^

* ND referred to no bacteria detected. All values are presented as mean ± standard deviation of triplicate. Different lowercase letters indicate statistically significant differences within each column (*Salmonella* or phage counts) (*p* < 0.05).

**Table 4:** *Salmonella* and phage counts after treatment on rice husks.

Time (Days)	*Salmonella* Count (log CFU/g)		Phage Count (log PFU/g)
	Control	Phage	
0	3.6 ± 0.1 ^a^	3.4 ± 0.5 ^c^	6.3 ± 0.3 ^c^
1	3.5 ± 0.1 ^a^	3.3 ± 0.9 ^c^	6.4 ± 0.2 ^c^
3	4.9 ± 0.3 ^b^	2.3 ± 0.3 ^b^ *	6.9 ± 0.6 ^c^
5	6.5 ± 1.1 ^d^	2.2 ± 0.4 ^b^ *	5.4 ± 0.4 ^b^
7	5.9 ± 1.0 ^c^	2.0 ± 0.5 ^b^ *	5.9 ± 0.6 ^bc^
14	5.1 ± 0.9 ^d^	1.7 ± 0.3 ^a^ *	4.6 ± 0.1 ^a^

All values are presented as mean ± standard deviation of triplicate. Within each column (*Salmonella* counts in the control and phage treatment, and phage counts), different lowercase letters indicate statistically significant differences (*p* < 0.05). The asterisk (*) denotes a significant difference (*p* < 0.05) between the control and phage treatment at the same time point.

**Table 5:** *Salmonella* and phage counts on chicken meat after treatment.

Time (Days)	*Salmonella* Count (log CFU/g)		Phage Count (log PFU/g)
	Control	Phage	
0	3.2 ± 0.7 ^a^	3.3 ± 0.6 ^c^	6.5 ± 0.7 ^c^
1	4.9 ± 0.1 ^a^	4.1 ± 0.3 ^c^ *	6.9 ± 0.3 ^c^
3	6.5 ± 0.4 ^b^	5.8 ± 0.4 ^b^ *	7.4 ± 0.2 ^c^
5	7.4 ± 0.1 ^d^	5.7 ± 0.1 ^b^ *	6.4 ± 0.5 ^b^

All values are presented as mean ± standard deviation of triplicate. Within each column (*Salmonella* counts in the control and phage treatment, and phage counts), different lowercase letters indicate statistically significant differences (*p* < 0.05). The asterisk (*) denotes a significant difference (*p* < 0.05) between the control and phage treatment at the same time point.

**Table 6:** *Salmonella* and phage counts after treatment of non-food material surfaces.

Time (h)	*Salmonella* Count (log CFU/cm^2^)					
	Ceramics		PVC		Stainless Steel	
	Control	Phage	Control	Phage	Control	Phage
0	3.1 ± 0.5 ^a^	3.2 ± 0.4 ^a^	3.0 ± 0.4 ^a^	3.1 ± 0.3 ^a^	3.2 ± 0.3 ^a^	3.2 ± 0.6 ^a^
24	3.0 ± 0.4 ^a^	1.7 ± 0.5 ^b^ *	3.0 ± 0.1 ^a^	2.4 ± 0.1 ^b^ *	3.3 ± 0.1 ^a^	2.0 ± 0.7 ^b^ *

All values are presented as mean ± standard deviation of triplicate. Within each column of *Salmonella* counts (control and phage treatment), different lowercase letters indicate statistically significant differences (*p* < 0.05). The asterisk (*) denotes a significant difference (*p* < 0.05) between the control and phage treatment at the same time point.

## Data Availability

The research data supporting the findings of this study are available within the article and/or its Supplementary Materials. The nucleotide sequence of *Salmonella* phage WP110 has been deposited in the GenBank database under accession numbers PX354330.

## Supplementary Materials

The following supporting information can be downloaded at: https://www.mdpi.com/article/10.3390/antibiotics15080747/s1, Figure S1: Phage terminal analysis using PhageTerm shows that the genome of WP110 is not permuted with redundant ends. The packaging class is Long Direct Terminal Repeats (DTR); Figure S2: Predicted structure quality assessment of WP110-gp057 using ColabFold-AlphaFold2. (A) Predicted aligned error (PAE) plots of the five gp057 models ranked by confidence. (B) Predicted local distance difference test (pLDDT) scores per residue for the five ranked models. (C) Multiple sequence alignment (MSA) coverage of gp057 shows sequence coverage (black line), while the colour gradient indicates the sequence identity to the query; Figure S3: Amino acid sequence alignment between EndoT5 endolysin and WP110-gp057 through Clustal Omega. Red frames represent the active amino acid residues involved in the hydrolysis of peptidoglycan; Figure S4: The 3D structure of the docking between EndoT5 endolysin (PDB: 2mxz) and peptidoglycan by SwissDock. (A) The overview shows the possible configuration of a peptidoglycan (magenta) close to the active sites of the EndoT5 endolysin. (B) Close-up view demonstrates how possible a pepti-doglycan (magenta) will bind to the groove of the active site. Yellow dashes represent distance (Å) between the selected atoms; Table S1: Genome Annotation of phage WP110; Table S2: VIRIDIC result; Table S3: SwissDock protein–ligand docking scores for selected models of WP110-gp057 with a molecule of peptidoglycan.
