# Supplementary material for: Comprehensive Characterization of a Novel Broad-Host-Range Lytic Salmonella Phage WP110 and Its Biocontrol Potential Across the Broiler Value Chain
Source: Antibiotics (Basel). 2026 Jul 31;15(8):747. doi: 10.3390/antibiotics15080747 (PMC13510199; doi:10.3390/antibiotics15080747)
Supplement: Supplementary file 1 [file antibiotics-15-00747-s001.zip › Supplementary data.pdf]

Supplementary Data

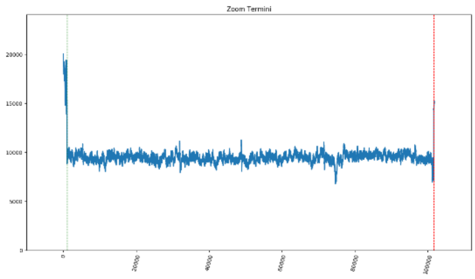

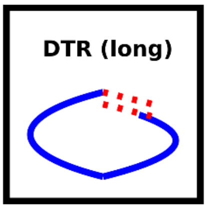

PhageTerm Method

Ends	Left (red)	Right (green)	Permuted	Orientation	Class	Type
Redundant	110098	9428	No	NA	DTR (long)	T5

Direct Terminal Repeats: 9547 bp

Strand	Location	T	pvalue
	110098	0.58	3.67e-68
	8939	0.07	8.28e-221
+	110100	0.06	1.19e-05
	8938	0.06	1.28e-172
	110096	0.04	1.14e-01
	9428	0.39	6.27e-80
	57393	0.11	5.90e-29
-	80045	0.05	8.49e-12
	40476	0.05	4.80e-08
	57392	0.05	5.56e-10

T (Start. Pos. Cov. / Whole Cov.)

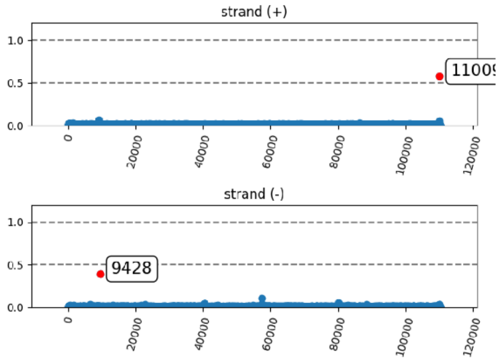

Figure S1. **Phage terminal analysis using PhageTerm shows that the genome of WP110 is not permuted with redundant ends**. The packaging class is Long Direct Terminal Repeats (DTR).

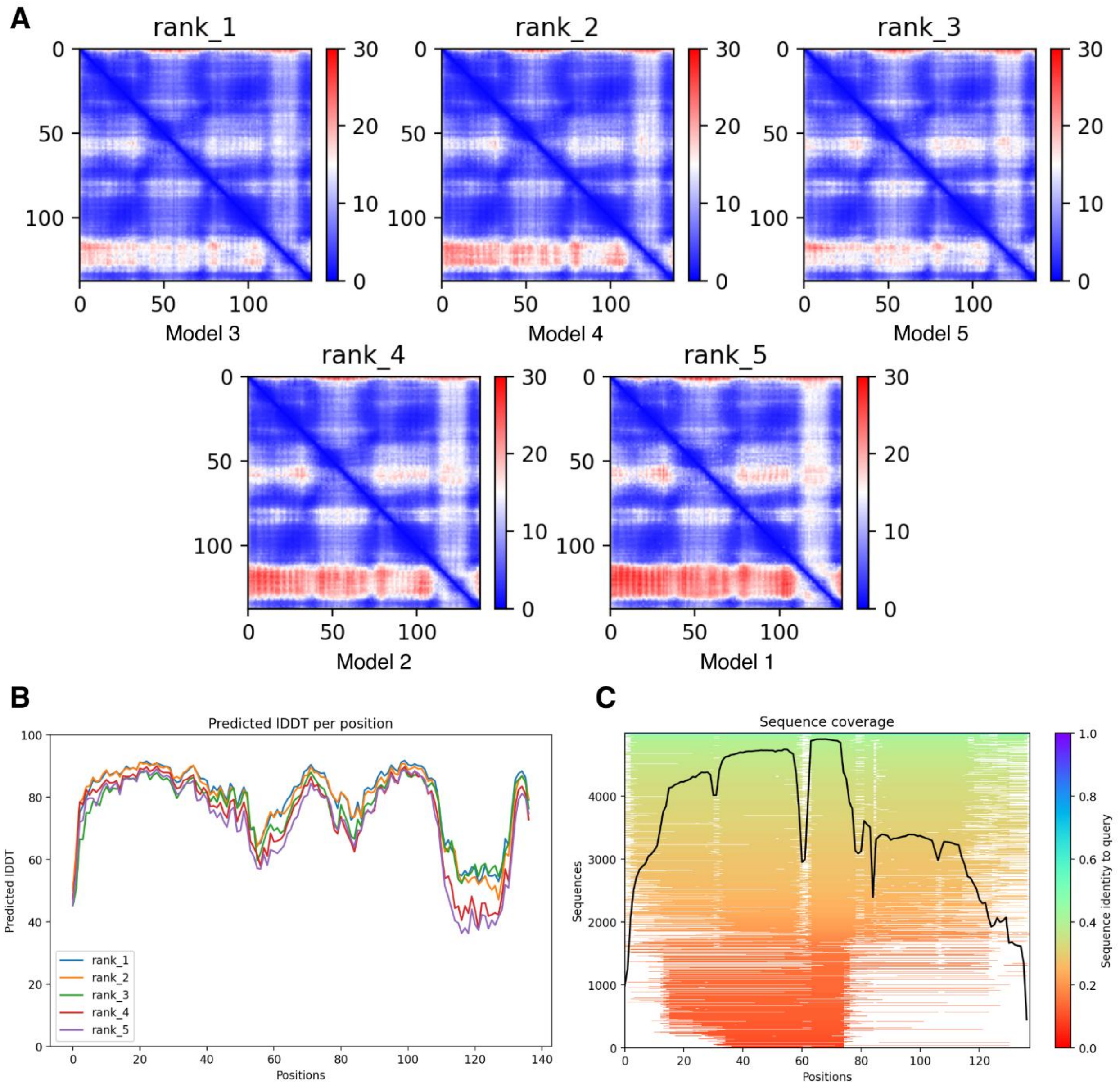

Figure S2. Predicted structure quality assessment of WP110-gp057 using ColabFold-AlphaFold2. (A) Predicted aligned error (PAE) plots of the five gp057 models ranked by confidence. **(B)** Predicted local distance difference test (pLDDT) scores per residue for the five ranked models. **(C)** Multiple sequence alignment (MSA) coverage of gp057 shows sequence coverage (black line), while the colour gradient indicates the sequence identity to the query.

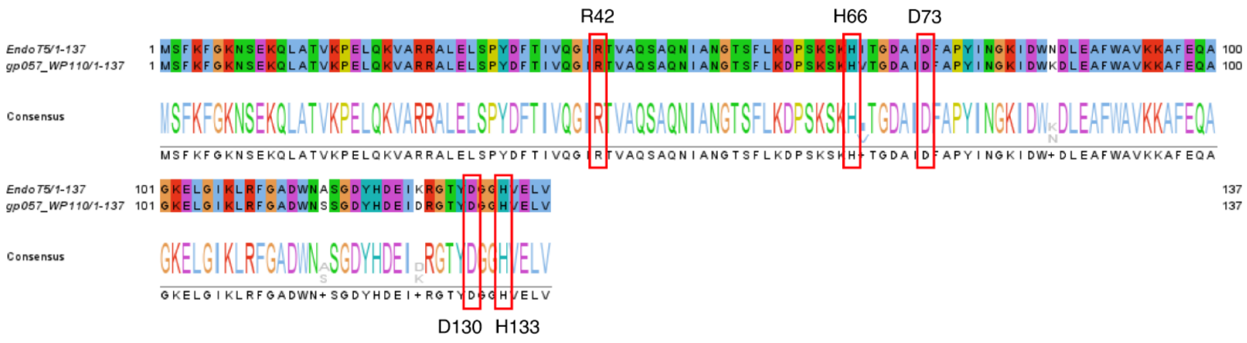

Figure S3. Amino acid sequence alignment between EndoT5 endolysin and WP110-gp057 through Clustal Omega. Red frames represent the active amino acid residues involved in the hydrolysis of peptidoglycan.

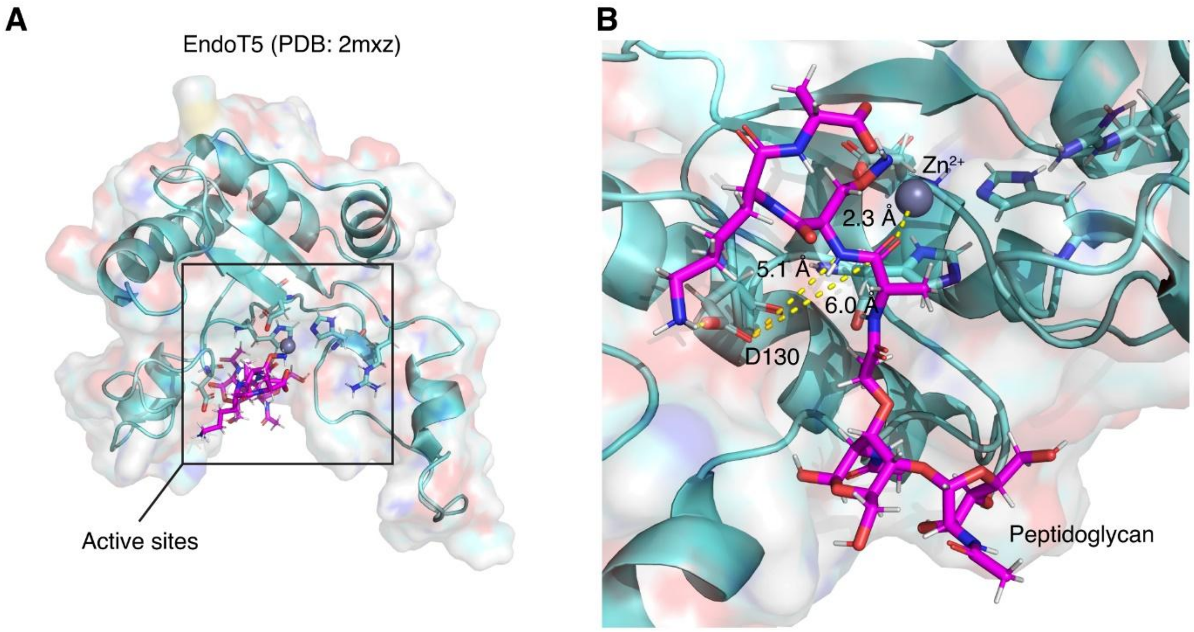

Figure S4. The 3D structure of the docking between EndoT5 endolysin (PDB: 2mxz) and peptidoglycan by SwissDock. (A) The overview shows the possible configuration of a peptidoglycan (magenta) close to the active sites of the EndoT5 endolysin. **(B)** Close-up view demonstrates how possible a peptidoglycan (magenta) will bind to the groove of the active site. Yellow dashes represent distance (Å) between the selected atoms.

Table S3. SwissDock protein–ligand docking scores for selected models of WP110-gp057 with a molecule of peptidoglycan.

Model	SP-dG (kcal/mol)	Polar	Nonpolar	Inter (kcal/mol)	Polar15	RMSD (Å)
26	-6.8692	43.3888	-58.5048	-15.1161	6.5783	17.1319
28	-7.1420	40.7943	-59.7102	-18.9159	1.4813	16.6976
52	-6.9184	33.3733	-52.5457	-19.1724	-2.4857	15.5188
53	-6.6524	63.5789	-69.2753	-5.6965	26.0929	15.3321
60	-7.5482	51.9569	-71.2262	-19.2693	6.7091	17.0001
